# Ceruloplasmin Plays a Neuroprotective Role in Cerebral Ischemia

**DOI:** 10.3389/fnins.2018.00988

**Published:** 2019-01-08

**Authors:** Fari Ryan, Juan G. Zarruk, Lena Lößlein, Samuel David

**Affiliations:** Centre for Research in Neuroscience, The Research Institute of the McGill University Health Centre, Montreal, QC, Canada

**Keywords:** stroke, ferroxidase, iron, oxidative damage, ferritin

## Abstract

Ceruloplasmin (Cp) is a ferroxidase that also plays a role in iron efflux from cells. It can thus help to regulate cellular iron homeostasis. In the CNS, Cp is expressed as a membrane-anchored form by astrocytes. Here, we assessed the role of Cp in permanent middle cerebral artery occlusion (pMCAO) comparing wildtype and Cp null mice. Our studies show that the lesion size is larger and functional recovery impaired in Cp null mice compared to wildtype mice. Expression of Cp increased ninefold at 72 h after pMCAO and remained elevated about twofold at day 14. We also assessed changes in mRNA and protein expression of molecules involved in iron homeostasis. As expected there was a reduction in ferroportin in Cp null mice at 72 h. There was also a remarkable increase in DMT1 protein in both genotypes at 72 h, being much higher in wildtype mice (19.5-fold), that then remained elevated about twofold at 14 days. No difference was seen in transferrin receptor 1 (TfR1) expression, except a small reduction in wildtype mice at 72 h, suggesting that the increase in DMT1 may underlie iron uptake independent of TfR1-endosomal uptake. There was also an increase of ferritin light chain in both genotypes. Iron histochemistry showed increased iron accumulation after pMCAO, initially along the lesion border and later throughout the lesion. Immunofluorescence labeling for ferritin (a surrogate marker for iron) and GFAP or CD11b showed increased ferritin in GFAP+ astrocytes along the lesion border in Cp null mice, while CD11b+ macrophages expressed ferritin equally in both genotypes. Increased lipid peroxidation assessed by 4HNE staining was increased threefold in Cp null mice at 72 h after pMCAO; and 3-nitrotyrosine labeling showed a similar trend. Three key pro-inflammatory cytokines (IL-1β, TNFα, and IL-6) were markedly increased at 24 h after pMCAO equally in both genotypes, and remained elevated at lower levels later, indicating that the lack of Cp does not alter key inflammatory cytokine expression after pMCAO. These data indicate that Cp expression is rapidly upregulated after pMCAO, and loss of Cp results in dysregulation of iron homeostasis, increased oxidative damage, greater lesion size and impaired recovery of function.

## Introduction

Dysregulation of cellular iron homeostasis is considered to contribute to neural damage after ischemic stroke (Millerot-Serrurot et al., 2008; Texel et al., 2011; Garcia-Yebenes et al., 2012). Iron plays an important role in many physiological processes by catalyzing a variety of enzymatic reactions (Rouault and Tong, 2005). The reason for these various functions is the redox active nature of iron, which can transition between the ferrous (Fe2+) and ferric (Fe3+) states. Divalent ferrous iron (Fe2+) can react with hydrogen peroxide in the Fenton reaction to catalyze the formation of free radicals, which cause oxidative damage (Crielaard et al., 2017). Therefore, the levels of cellular iron needs to be tightly regulated via a complex system of interactions involving several cell surface and intracellular proteins (Hentze et al., 2004; Crielaard et al., 2017).

Ceruloplasmin (Cp) is a ferroxidase that safely converts ferrous (Fe2+) iron to its ferric (Fe3+) form (Williams et al., 1974; Calabrese et al., 1989). The soluble form of Cp is expressed by the liver and found in serum (Gitlin, 1988). We identified a glycosylphosphatidylinositol-anchored form of Cp (GPI-Cp) in the central nervous system (CNS) that is expressed by astrocytes (Patel and David, 1997; Patel et al., 2000; Jeong and David, 2003). We have also reported that GPI-Cp is expressed abundantly by meningeal cells that surround the brain and spinal cord (Mittal et al., 2003). The choroid plexus located in the ventricular spaces in the CNS, and which produces cerebrospinal fluid, expresses the soluble form of Cp (Patel and David, 1997). We provided early evidence that GPI-Cp partners with the iron efflux transporter ferroportin to regulate iron efflux from astrocytes (Jeong and David, 2003). We and others have reported that Cp gene knockout mice show disruption of iron homeostasis in the CNS and other tissues such as the liver (Harris et al., 1999; Patel et al., 2002) and lead to iron accumulation in the CNS with age (>18 months of age), resulting in free-radical mediated neuronal loss (Patel et al., 2002; Jeong and David, 2006). Other work also indicates that the absence of GPI-Cp results in the internalization and degradation of the iron exporter, ferroportin (Fpn), from the cell surface, which results in iron accumulation (De Domenico et al., 2007). Iron accumulation in the CNS and other organs, which increases with age is a characteristic feature of humans with aceruloplasminemia, which results from mutations in the Cp gene (Harris et al., 1995; Morita et al., 1995; Yoshida et al., 1995; Xu et al., 2004). We have also reported a slow accumulation of iron in the brain in Cp null mice with age (>18 months of age) (Patel et al., 2002; Jeong and David, 2006).

Mechanisms that lead to iron accumulation in cells in the CNS in different neurological conditions are not fully understood but can give rise to oxidative damage and neurodegeneration. In CNS injury and disease, increased iron loading of cells occurs either when dying cells release their intracellular iron content into the extracellular space (as in cell necrosis or physical damage) and/or are taken up by microglia and macrophages (phagocytosis) as occurs in ischemic stroke. Increased iron load in the CNS also occurs acutely after hemorrhage and uptake of red blood cells by phagocytes, as occurs in hemorrhagic stroke. In addition, dysregulation of molecules involved in maintaining cellular iron homeostasis can also result in iron accumulation, i.e., increased iron uptake or reduce iron efflux. The extent of iron-mediated free-radical damage will depend on how well excess iron is taken up and safely sequestered in ferritin or exported/effluxed out of the cells (Finazzi and Arosio, 2014). Moreover, in CNS disorders such as multiple sclerosis (MS), iron accumulation is seen in microglia that eventually appear to become dystrophic and undergo cell death (Hametner et al., 2013), which could lead to further cycles of iron uptake and cell death. We have reported recently, that iron accumulation in macrophages in a mouse model of MS [experimental autoimmune encephalomyelitis (EAE)] may be due to hepcidin-mediated internalization of Fpn (Zarruk et al., 2015).

In the present study, we examined the role of Cp in ischemic stroke using wildtype and Cp null mice. We examined the role of Cp on lesion size and functional recovery, as well as changes in the expression of various molecules involved in iron homeostasis in the ischemic brain. We also studied the extent of iron deposition, free radical damage and changes in the expression of inflammatory cytokines. This work suggests that Cp plays a significant role in functional recovery and tissue neuroprotection after permanent cerebral ischemia.

## Materials and Methods

### Induction of Permanent Middle Cerebral Artery Occlusion (pMCAO)

Male Cp-/- and Cp+/+ mice on a C57BL/6 background, 8–12 weeks of age, were used. Ceruloplasmin knockout mice were generated in our lab several years ago as previously described (Patel et al., 2002). All surgical procedures were approved by the Animal Care Committee of the Research Institute of the McGill University Health Centre and followed the guidelines of the Canadian Council on Animal Care, and the ARRIVE guidelines for reporting animal research (Kilkenny et al., 2010). The pMCAO surgery was done as described previously (Zarruk et al., 2012) and is a variant of an earlier model (Chen et al., 1986). Briefly, animals were deeply anesthetized with isoflurane in O_2_ (0.5–1 L/min; inhalation) during the entire procedure. During surgery, body temperature was maintained at 37.0 ± 0.5°C using a homeothermic system with a rectal probe (Harvard apparatus). A small craniotomy was made over the region of the trunk of the left middle cerebral artery (MCA) above the rhinal fissure. The pMCAO was done by ligating the trunk of the MCA just before its bifurcation between the frontal and parietal branches using a 9-0 suture. Complete interruption of blood flow was confirmed under visual inspection under the operating microscope. In addition, the left common carotid artery was then occluded. After surgery, animals were returned to their cages with free access to water and food. Spontaneous mortality was not observed after the surgical procedure.

### Infarct Volume

Nissl stained tissue sections (30 μm) spanning the entire lesion were assessed to estimate the infarct volume using the ImageJ software. To calculate the infarct volume, two measurements were taken: the whole ipsilateral hemisphere and the ipsilateral hemisphere excluding the lesion. The 2 values were used to calculate the % of infarcted hemisphere. This is done to accurately estimate the lesion in each animal and to exclude any variations in brain size or distortions due to histological preparation. These values were used for statistical analysis.

### Functional Assay

The “adhesive-tape removal” assay was used to assess functional recovery. This test assesses the integrity of motor and sensory function. It was originally described for rats and then adapted to mice (Bouet et al., 2009; Zarruk et al., 2011). In brief, a small piece of adhesive tape is placed over the hairless parts of the forepaw (pads, thenar, and hypothenar). This is repeated on both sides. Habituation and training was done for 5 days before surgery, each time alternating the order in which the tape was placed (left or right paw). The test was done on 1, 2, 7, and 14-days post-surgery (*n* = 13–15 mice per group). Three trials were done per day per mouse and the mean of each day was calculated. On the evaluation day, after the adhesive tape was placed, the mice were placed back into the evaluation box/cage, and the time taken to remove the tape is measured. Sham surgery animals were used as controls. The asymmetry between the right and left sides was calculated by dividing the time taken to remove the tape on the affected side (right) versus the non-affected side (left). The higher the number meant greater disability which is explained because ischemic lesions will affect the sensorimotor abilities of the mice on the contralateral side, impairing their ability to feel and/or remove the tape.

### Collection of Tissue for mRNA and Western Blotting

Under deep anesthesia the mice were perfused via the heart with phosphate buffered saline and the brain rapidly exposed and removed; and the cortex dissected out and rapidly frozen in liquid nitrogen. The most rostral and caudal parts of the brain (1-mm thick) were excluded to avoid regions that lack the infarct. The ipsi and contralateral sides of the cerebral hemisphere were taken for analysis. Samples were taken for mRNA and protein at different time intervals as indicated in the results section.

### Quantitative Real Time Polymerase Chain Reaction (qPCR)

Total RNA was extracted using RNeasy Mini Kit (Qiagen, Mississauga, ON, Canada) following manufacturer’s instructions. cDNA was reverse transcribed with the Qiagen Quantinova Kit (Cat. No. 205411) and amplified using an ABI OneStep cycler (Applied Biosystems) using specific primer pairs as indicated in Table 1. GAPDH was used as an internal control gene. The results were quantified using the ΔΔCT method following standardization relative to GAPDH (Livak and Schmittgen, 2001).

**Table 1:** Primer sequences used for qPCR.

Gene	Forward sequence	Reverse sequence
Ceruloplasmin	ACATTGCTGCTGAGG AGGTCATCT	TGTTCCTCATCAGGG CCTCTTTGT
Ferroportin	AACCAGAGTCACTG TCATCAGCCA	TCGGCCCAAGTCA GTGAAGGTAAA
DMT1	TGAATCGGGCGAA TAAGCAGGA	TCAGCAAAGACGGC AACGACAA
Ferritin HC	TAAAGAACTGGGTGA CCACGTGAC	AAGTCAGCTTAGC TCTCATCACCG
Ferritin LC	GGCTTTCCAGGAAG TCACAGAGAT	TGGCCATGGAG AAGAACCTGAATC
TfR1	AAACTGGCTGAAACG GAGGAGACA	GCTGCTTGATGGTG TCAGCAAACT
TNF-α	TTGCTCTGTGAAGGG AATGG	GGCTCTGAGGAGT AGACAATAAAG
IL-1β	ATGGGCAACCACTTA CCTATTT	GTTCTAGAGAGT GCTGCCTAATG
IL-6	CTTCCATC CAGTTGCCTTCT	CTCCGACTTGTGAAG TGGTATAG
IL-10	ACAGCCGGGAAGACA ATAAC	CAGCTGGTCCTTTGT TTGAAAG
GAPDH	GGAGAAACCTGCCAA GTATGA	TCCTCAGTGTAGCCC AAGA
		


### Western Blotting

Brain tissue was obtained after intracardiac perfusion with PBS as described above. Total protein was extracted with 1% Nonidet P-40 (Sigma), 1% sodium deoxycholate (BDH Chemicals), 2% SDS, 0.15 M sodium phosphate (pH 7.2), and 2 mM EDTA, containing a mixture of protease inhibitors (Roche Diagnostics) as described previously (Jeong and David, 2003). Protein concentrations were estimated using the Bio-Rad DC protein assay following manufacturer’s instructions (Cat. No 500-0121). Then 25 μg of proteins per sample were separated by sodium dodecyl sulfate polyacrylamide electrophoresis (SDS–PAGE) gels 4–12% (Novex, life Technologies), transferred to polyvinylidene fluoride membrane (PVDF) (Millipore, Billerica, MA). Membranes were blocked in 5% milk in 0.05% PBS Tween-20 and incubated overnight at 4°C with the following primary antibodies: rabbit anti-ferritin (1:500; Sigma-Aldrich Inc.; F5012), rabbit anti-DMT1 (1:1000; Alpha Diagnostics), mouse anti-TfR1 (1:1000; Invitrogen; 13–6800), rabbit anti-Cp (1:1000 DakoCytomation; Q 0121), or rabbit anti-Fpn (1:300 Alpha Diagnostic; MTP11-A). Blots were washed and incubated with horseradish peroxidase-conjugated IgG (1:10,000 –100,000; Jackson ImmunoResearch, West Grove, PA, United States) and visualized with enhanced chemiluminescence (PerkinElmer). Equal loading of proteins was estimated by re-probing the membranes with rabbit anti-β-actin antibodies (1:1000; Sigma-Aldrich). The blots were quantified using ImageJ software (version 1.48r NIH-USA).

### Immunofluorescence and Confocal Microscopy

Mice were perfused with phosphate buffer saline followed by 4% paraformaldehyde in 0.1 M phosphate buffer. After post-fixation in the same fixative for 24 h, the tissue was cryoprotected in 30% sucrose and 14 μm thick coronal sections of the brain cut with a cryostat and used for the immunofluorescence labeling. Tissue sections were incubated with 0.3% Triton X-100 (Sigma-Aldrich), 5% normal goat serum (Jackson ImmunoResearch Inc.), 2% ovalbumin (Sigma-Aldrich) in phosphate buffer for 3 h room temperature (RT) to block non-specific binding of antibodies. Sections were then incubated overnight at 4°C with primary antibodies against ferritin (1:200). These primary antibodies were combined with the following cell type specific antibodies: rat anti CD11b (for macrophages/microglia; 1:200; Serotec; MCA711), guinea pig anti-glial acidic protein (GFAP) (for astrocytes; 1:500; Synaptic Systems cat No. 173004). Sections were washed in 0.05% PBS-Tween-20 and incubated with appropriate fluorescent-conjugated secondary antibodies, anti-rabbit Alexa Fluor 488, anti-rat Alexa Fluor 568 or anti-guinea pig Alexa Fluor 568 (all 1:500, Invitrogen). Tissue sections were viewed with a confocal laser scanning microscope (FluoView FV1000, Olympus) and micrographs taken with the FV10-ASW 3.0 software (Olympus). For comparing between groups, the same setting was applied in all images of each immunostaining. The quantification of the double labeling of GFAP and ferritin at 72 h after pMCAO was assessed using the Image J software to estimate Pearson’s correlation coefficient as described previously (Dunn et al., 2011).

### Diaminobenzidine-Enhanced Turnbull’s Blue Staining for Iron

Turnbull’s blue iron histochemistry was done to detect ferric and ferrous iron as previously described (Meguro et al., 2007). Briefly, at different times after pMCAO mice were perfused with 0.1 M phosphate buffer followed by 4% paraformaldehyde. The brains were dissected out, and cryoprotected in 30% sucrose and 14 μm sections cut with a cryostat (Leica CM 3050 S). Sections were incubated in 10% ammonium sulfide for 90 min at RT, washed with distilled water (dH_2_O) and incubated for 30 min in a solution of 20% potassium ferricyanide (Sigma-Aldrich) and 1% hydrochloric acid. Sections were then washed in double distilled water and endogenous peroxidase blocked for 60 min at room temperature in 99 ml PBS + 0.01 M NaN3 (65 mg) + 1 ml 30% H_2_O_2_. After washes in 0.1 M phosphate buffer, iron staining in the tissue sections was visualized by incubation with 0.025% 3,3′ diaminobenzidine with 0.005% H_2_O_2_ in 0.1 M phosphate buffer (20 min at RT). The reaction was stopped with tap water and sections were counterstained with Mayer’s hemalum solution (EMD Millipore, 1.09249.1000) and mounted with Entellan (Electron Microscopy Sciences). For quantification of iron labeled cell at 72 h post-pMCAO, the number of labeled cells were counted and estimated per μm^2^. Direct cell counts of iron labeled cells could not be done at 14 days because the cells were densely packed in the lesion. Therefore, for quantification of iron staining at 14 days, we used densitometry to estimate the integrated density as follows: microscope images were prepared and analyzed with Image J (version 1.48r NIH-USA). The background was first subtracted with a rolling ball of 200 pixels. Staining intensity was then quantified using the densitometry 1 channel plug-in with a tolerance of 100 and the mean of integrated density was calculated for each animal and each group and plotted on the *y*-axis.

### Immunohistochemistry for 4HNE and 3-NT

Brain cryostat sections (14 μm thick) from 4% paraformaldehyde perfused-fixed mice, 72 h post-pMCAO were used for DAB immunohistochemistry for 4HNE (4-Hydroxynonenal) and 3-NT (3-Nitrotyrosine) as described previously (Zarruk et al., 2015). In brief, endogenous peroxidase was blocked with 0.2% H_2_O_2_ in PBS for 30 min at RT. Then non-specific binding was blocked for 3 h at RT in blocking solution (3% Triton, 5% normal goat serum, in PBS) with avidin D solution (Vector laboratories; SP2001) followed by overnight incubation with primary rabbit anti-4HNE antibody (1:300; Abcam; AB46545) or rabbit anti-3NT antibody (1:500; Invitrogen; A21285), with the biotin solution (Vector laboratories; SP2001). The Avidin/Biotin blocking kit (Vector) was used as an extra step to block non-specific avidin/biotin binding. Sections were washed in 0.05% PBS-Tween and incubated for 1 h at RT with secondary goat anti-rabbit biotinylated (1:1000; Jackson ImmunoResearch), washed and incubated with avidin peroxidase 1 h (RT) (1:100; Sigma #A- 2693151). Labeling was visualized with 3,3′ diaminobenzidine-tetrahydro chloride using the ImmPACT DAB peroxidase substrate kit according to manufacturer instructions (Vector; SK-4105) and counterstained with Mayer’s hemalum. The number of cells per μm^2^ was quantify in each group from 10x images taken with a Zeiss Axioskop II light microscope using Image J (version 1.48r NIH-USA).

### Statistical Analyses

All types of analyses were carried out blind, so the evaluator was not aware of the groups. Data are shown as mean ± Standard Error of the Mean (SEM). Two-way ANOVA followed by Bonferroni’s *post hoc* test was used to analyze the results from “adhesive tape removal” test at different time points. The colocalization of GFAP and ferritin was quantified using Pearson’s colocalization coefficient (Dunn et al., 2011) with the ImageJ colocalization Threshold plug-in. A zero value is given for the pixels below the thresholds. That means that all the pixels which have intensities above the two thresholds have greater than zero correlation, and the pixels below the thresholds have none or negative correlated intensities. Therefore, the closest the value is to 1 the better the colocalization (1 = full colocalization, 0 = no colocalization). Mann–Whitney non-parametric test was used to compare two groups. A value of *p* ≤ 0.05 was considered significant.

## Results

### Decreased Functional Recovery in Cp Null Mice After pMCAO

**FIGURE 1:**
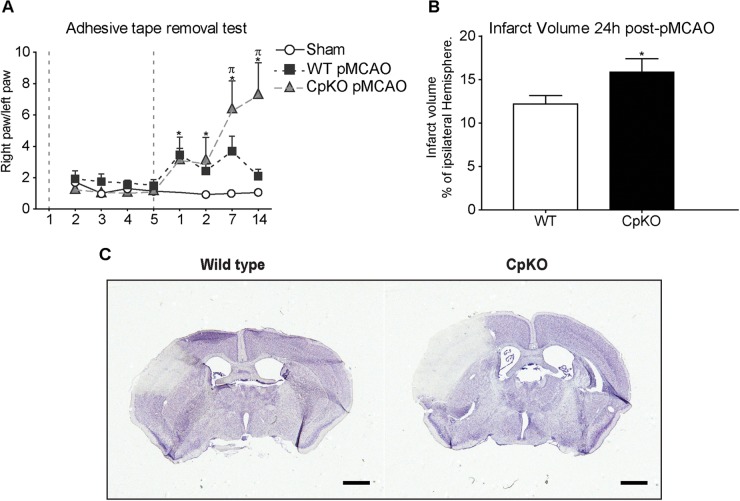
**(A)** Graph showing functional recovery assessed with the “adhesive-tape removal” test. Mice were trained for 5 days prior to the surgery for pMCAO (period indicated between the two vertical dashed lines). At days 1 and 2 after pMCAO wildtype and Cp KO mice both show similar deficits. Wildtype mice recover to normal values by day 14, while CpKO mice show increasing functional deficits at 7 and 14 days. The sham animals show no deficits. **(B)** Cp null mice show significantly larger lesion size as compared to wildtype mice at 24 h after pMCAO. **(C)** Nissl stained sections showing the ischemic lesion in wildtype and Cp KO mice 24 h after pMCAO. *n* = 13–15 per group in **A**; and 9 per group in **B**. Two-way ANOVA was used to analyze results in A, followed by Bonferroni’s *post hoc* test. ^∗^*p*-value ≤ 0.05 compared to sham-control group; π*p*-value ≤ 0.05 compared to wild-type group. Mann–Whitney test was used to compare WT vs. CpKO in B. ^∗^*p*-value ≤ 0.05; scale bar = 100 μm.

We first assessed functional recovery after pMCAO using the “adhesive-tape removal” test. In this test, a small piece of adhesive tape is stuck on the paw of each forelimb, and the time taken by the mouse to remove the tape is estimated. This test showed that Cp KO mice took a longer time to remove the tape as compared to wildtype mice (Figure 1A). Interestingly, the Cp KO and wildtype mice showed similar levels of deficit at 1 and 2 days after pMCAO. However, at 7 and 14 days, the two groups diverged with the Cp null mice showing increasingly worse deficits, while the wildtype mice showed recovery to normal values at 14 days. The latter may be due to neural plasticity in wildtype mice, which appears to be impaired in Cp null mice, possibly due to free radical mediated secondary damage to neurons (e.g., dendritic spine formation) and glia (microglia and astrocytes). For example, pruning of synaptic arbors by microglia is required for establishment of proper function (Stephan et al., 2012). Astrocytes are also required for recycling of neurotransmitters and the proper functioning of synapses (Allen, 2014). We have reported previously that Cp null mice show changes in CNS glia with reduction of astrocytes with age (Jeong and David, 2006). Sham controls did not show any deficits.

### Infarct Size Is Larger in Cp Null Mice

We next determined the infarct size using Nissl stained tissue sections obtained from mice 24 h and 14 days after pMCAO. The ImageJ software was used to measure the total infarct volume and presented as a percentage of the ipsilateral hemisphere (Figure 1B). This analysis showed a significant increase in the size of the lesion in Cp null mice 24 h after pMCAO (17.5 mm^3^) as compared to wildtype mice (15.8 mm^3^) (Figures 1B,C). At 14 days post-pMCAO there was a trend to an increase in the Cp null mice, but these differences were not statistically significant (data not shown). This may be because by 14 days there is marked shrinkage of the lesion leading eventually to a scar, making the differences less prominent.

### Changes in the Expression of Iron Homeostasis Proteins at the mRNA and Protein Levels

#### Ceruloplasmin

**FIGURE 2:**
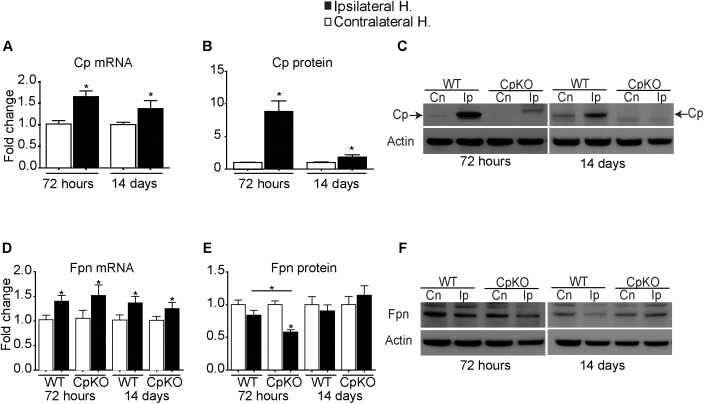
Changes in mRNA detected by q-PCR and protein expression detected by Western blot analysis of Cp **(A–C)** and Fpn **(D–F)** in the ipsilateral ischemic side as compared to the contralateral uninjured side at 72 h and 14 days after pMCAO. Changes in the ipsilateral ischemic side are represented as fold-change as compared to contralateral uninjured side. Changes in expression of Cp mRNA **(A)**, and protein **(B)** quantified from Western blots; a representative example of the Western blot for Cp is shown **(C)**. Note the marked increase in Cp protein at 72 h (8.8-fold) and which remains significantly elevated (twofold) at 14 days. **(C)** The weak bands seen on the ipsilateral side in the Cp null mice are non-specific bands that are not at the expected 135 kDa GPI-Cp band (arrows). Changes in Fpn mRNA **(D)** and protein **(E)** and representative Western blots **(F)**. Fpn is significantly reduced on the ipsilateral side in Cp null mice at 72 h. β actin was used as a loading control. *n* = 6 per group; ^∗^*p*-value ≤ 0.05.

In wildtype mice, there is a modest but significant increase in Cp mRNA expression in the ipsilateral ischemic hemisphere as compared to the uninjured contralateral hemisphere at both 72 h and 14 days after pMCAO (Figure 2A). Interestingly, Western blot analysis showed an 8.8-fold increase in Cp expression at 72 h after pMCAO and remained significantly elevated at about twofold at day 14 (Figures 2B,C). These findings suggest up-regulation of Cp expression at the protein level in ischemic stroke. This increase in Cp expression is in keeping with its protective role as a ferroxidase to reduce the toxic effects of ferrous iron and to export iron out of cells, particularly astrocytes in the CNS, and thus prevent intracellular iron accumulation.

#### Ferroportin

Cp partners with ferroportin (Fpn), the ubiquitously expressed iron efflux transporter, to export iron out of cells. We therefore assessed the expression of Fpn at the mRNA and protein levels. At the mRNA level there was a modest increase in Fpn expression on the ipsilateral ischemic cortex in both genotypes at 72 h and 14 days (Figure 2D). However, at the protein level there was about a 50% reduction in Fpn expression at 72 h in the ipsilateral side in Cp null mice versus the contralateral uninjured cortex (Figures 2E,F), as well as a significant reduction in the ipsilateral side in Cp null mice compared to wildtype mice at 72 h (Figures 2E,F). Our previous studies have shown that GPI-Cp is required for the stability of Fpn on the cell surface (De Domenico et al., 2007). In the absence of GPI-Cp, Fpn gets internalized and eventually degraded. This may account for the reduction of Fpn protein acutely in Cp null mice at 72 h. In addition, the partial reduction in Fpn protein may be because Cp is only expressed by astrocytes but no other cell types in the CNS (Patel and David, 1997). No differences in Fpn protein were seen in wildtype mice at 72 h and between any of the groups at 14 days post-pMCAO despite increases in Fpn mRNA on the ipsilateral side. Why the increase in mRNA levels on the ipsilateral side are not reflected in increase in protein levels in the Western blots is not known at present but may involve regulated internalization and degradation of the protein.

#### Transferrin Receptor 1 (TfR1) and Divalent Metal Ion Transporter 1 (DMT1)

**FIGURE 3:**
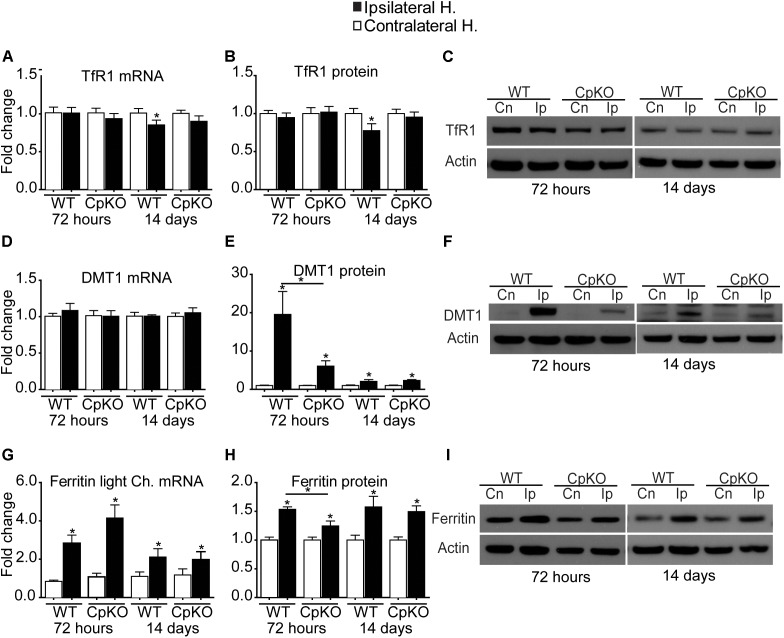
Changes in mRNA and protein expression of TfR1 **(A–C)**, DMT1 **(D–F)**, and ferritin **(G–I)** shown as fold-change in the ipsilateral ischemic side as compared to the contralateral uninjured side at 72 h and 14 days after pMCAO. No changes are seen in TfR1 mRNA **(A)** or protein **(B)** except for a small but significant reduction in the ipsilateral side in wildtype mice at 14 days. Representative Western blot shown in **C**. Changes in expression of DMT1 mRNA **(D)**, and protein **(E)** quantified from Western blots; representative Western blot shown in **F**. Note the marked increase in DMT1 protein on the ipsilateral side in wildtype (19.5-fold) and Cp null (sixfold) mice at 72 h, which remain elevated at twofold at 14 days in both genotypes. Changes in mRNA expression of ferritin light chain **(G)** and ferritin protein **(H)** and a representative Western blot **(I)**. Note the increase in mRNA and protein levels in the ipsilateral side in all groups. β actin was used as a loading control; note that the same blot was used to probe DMT1 **(F)** and Cp (Figure 2C) hence the actin blots for these are the same. *n* = 6 per group; ^∗^*p*-value ≤ 0.05.

There was a small but significant reduction in TfR1 mRNA and protein in the ipsilateral cortex in wildtype mice at 14 days post-ischemia. No other differences were seen (Figures 3A–C).

No differences were observed in the mRNA level of DMT1 after pMCAO in either genotype (Figure 3D). In contrast, Western blot analysis of tissue taken at 72 h post-pMCAO showed a large almost 19.5-fold increase in DMT1 protein in the ipsilateral ischemic cortex in wildtype mice and a smaller (sixfold) increase in the ipsilateral cortex in Cp null mice (Figures 3E,F). A smaller (twofold) significant increase in DMT1 protein continued to be detected in the ipsilateral cortex of both genotypes at 14 days (Figures 3E,F). DMT1 is known to be involved in release of endosomal iron taken up via the Tf-TfR1 complex. DMTI is also expressed on the surface of astrocytes (Jeong and David, 2003) and so may be involved in transferrin-independent iron uptake into cells. Our data showing no increases in TfR1 expression would suggest that the increase in DMT1 might contribute to direct uptake of iron into astrocytes after cerebral ischemia. It is also important to note that DMT1 protein expression was 3 times lower in Cp null mice compared to wildtype mice at 72 h (Figure 3E).

#### Ferritin

Ferritin is the iron storage protein in cells. Each molecule of ferritin consists of 24 heavy and light chain subunits that form a sphere in which iron is stored. Each molecule of ferritin can store up to 4,500 atoms of iron and so is a very efficient iron storage protein (Arosio and Levi, 2002, 2010). Excess iron is stored in ferritin. There is a significant increase at the mRNA level of ferritin light chain (the major iron storage form) in the ipsilateral hemispheres in both genotypes compared to the contralateral hemispheres. The mRNA increase at 72 h in wildtype mice is about threefold and in Cp null mice about fourfold (Figure 3G). In contrast, mRNA expression of ferritin heavy chain, which has ferroxidase activity, showed no difference after ischemia in either genotype, except for a small but significant increase in the ipsilateral side in Cp null mice at 72 h (data not shown). At the protein level in Western blots, the antibody does not distinguish between heavy and light chains, so the results are a summation of the levels of both chains. In the ipsilateral ischemic cortex there is a significant increase in ferritin protein at 72 h and 14 days in both genotypes (Figures 3H,I).

### Iron Histochemistry and Ferritin Immunofluorescence Labeling

**FIGURE 4:**
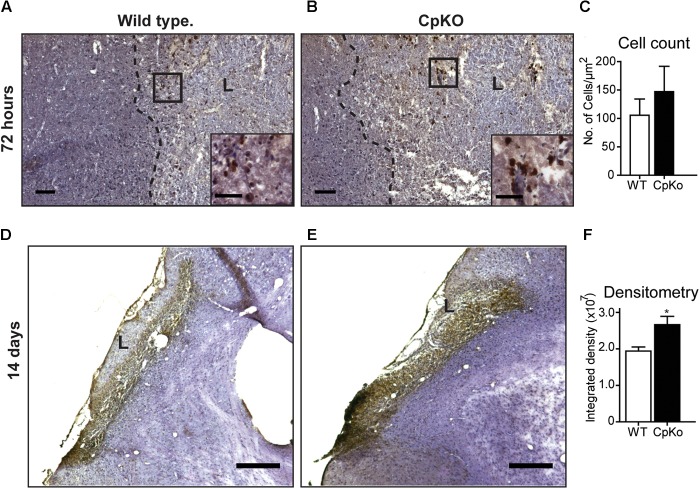
Iron histochemistry. Detected by the enhanced Turnbull’s staining of tissue sections taken 72 h **(A–C)** and 14 days **(D–F)** after pMCAO. At 72 h the cells labeled for iron (stained dark brown; see insets in **A** and **B**) are located in the lesion and distributed along the lesion border in both wildtype **(A)** and CpKO **(B)** mice; L = lesion; areas in the black squares in **A,B** are shown at higher magnification in the insets. **(C)** Graph shows that the number of iron+ cells is not significantly different in the two genotypes. At 14 days **(D,E)** the iron labeled cells are densely packed in the lesion (L) making it difficult to do cell counts. Densitometric analysis **(F)** showed higher levels of iron deposition in CpKO mice compared to wildtype mice at 14 days. All sections counterstained with Mayer’s hemalum stain. Scale bars in **A–E** = 100 μm, inset = 50 μm; *n* = 6 per group; ^∗^*p*-value ≤ 0.05.

To investigate further the changes in iron accumulation in ischemic stroke, we performed diaminobenzidine (DAB)-enhanced Turnbull’s blue iron histochemistry. These studies showed that iron labeled cells are seen in the lesion along the lesion border at 72 h after pMCAO in both wildtype and Cp knockout mice (Figures 4A,B). The cell counts of labeled cells did not differ between the two genotypes at 72 h (Figure 4C). However, at 14 days after pMCAO, there was a marked increase in iron staining throughout the lesion in both genotypes (Figures 4D,E). The labeled cells were densely packed in the lesion making it difficult to obtain cell counts. Densitometry analysis, however, showed a significant increase in Cp null mice compared to wildtype mice (Figure 4F).

**FIGURE 5:**
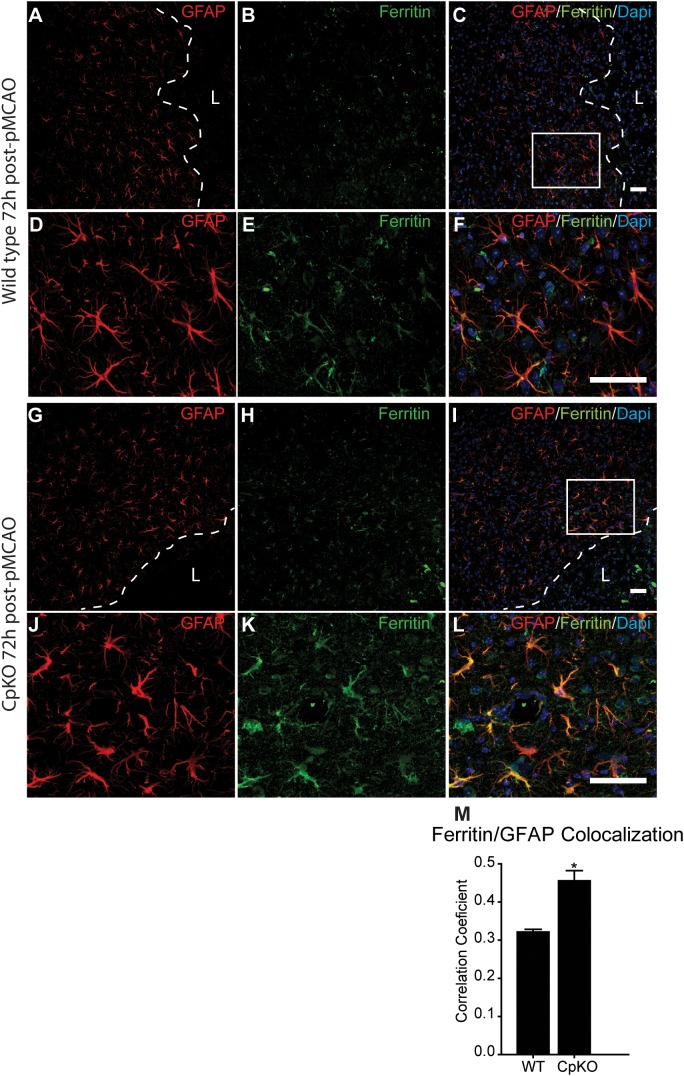
Immunofluorescence labeling for ferritin in astrocytes. Low magnification images of the border region of the infarct and the peri-infarct region in wildtype **(A–C)** and Cp KO **(G–I)** mice 72 h after pMCAO. Dotted line demarcates the lesion boundary and L marks the lesion site. The area in the white square in panels **C,I** are shown at higher magnification in panels **D–F** and **J–L**. Note increase in the number and intensity of the ferritin labeling in CpKO mice **(K)** as compared to the wildtype mice **(E)**. The number of astrocytes that are double labeled for ferritin is higher in the Cp KO **(K)** than in wildtype mice **(E)**. The merged images **(C,F,I,L)** show GFAP, ferritin and DAPI nuclear staining. **(M)** Graph showing the extent of double labeling of GFAP and ferritin estimated using Pearson’s correlation coefficient (*n* = 4). Scale bar = 50 μm, ^∗^*p*-value ≤ 0.05.

**FIGURE 6:**
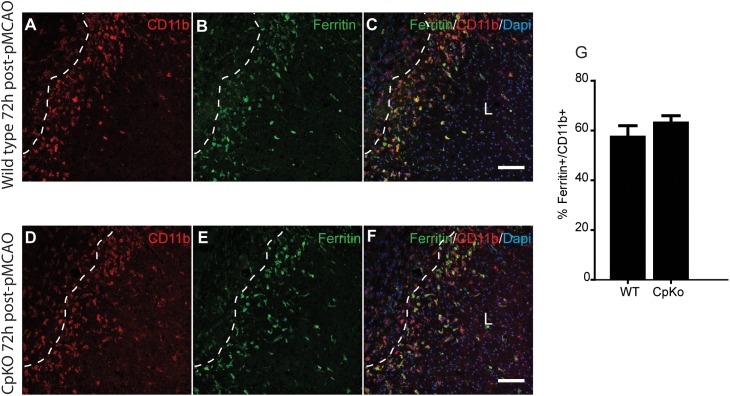
Immunofluorescence labeling for ferritin in macrophage/microglia. Double immunofluorescence labeling show that ferritin+/CD11b+ macrophage/microglia are located mainly in the lesion (L) along the lesion border in both wildtype **(A–C)** and CpKO **(D–F)** mice 72 h after pMCAO. Dotted line demarcates the lesion boundary. The merged images **(C,F)** show CD11b, ferritin and DAPI nuclear staining. Note that the number of CD11b+/ferritin+ doubled labeled cells are not significantly different in the two genotypes **(G)**. Scale bar = 100 μm.

Iron histochemistry is not compatible with immunostaining, therefore, to assess which cell types accumulate iron in the ischemic brain, we carried out double immunofluorescence labeling for ferritin, a good surrogate marker for iron, and co-labeled the sections with antibodies to GFAP (astrocytes) (Figure 5) and CD11b (macrophages/microglia) (Figure 6) at 72 h after pMCAO. The double labeling showed that in the ipsilateral ischemic cortex of Cp null mice, ferritin expression is more intense (Figures 5E,K) and co-localizes to significantly more GFAP-positive astrocytes as compared to wildtype mice (Figures 5F,L,M). Colocalization of ferritin and GFAP was quantified using Pearson’s correlation coefficient, as the two labels are in the cytoplasmic compartment (Figure 5M). These ferritin+ astrocytes are located in the intact CNS tissue along the border region of the infarct. Co-localization of ferritin in CD11b+ macrophages/microglia is seen in the ischemic lesioned tissue along the border region of the infarct (Figures 6A–F). The number of ferritin+/CD11b+ macrophages/microglia at 72 h post-pMCAO was similar in Cp null and wildtype mice (Figure 6G).

These results suggest that at 72 h post-pMCAO, the iron labeled cells seen by the Turnbull’s stain along the lesion border are likely to include both astrocytes and macrophage/microglia.

### Changes in Markers of Oxidative Stress

**FIGURE 7:**
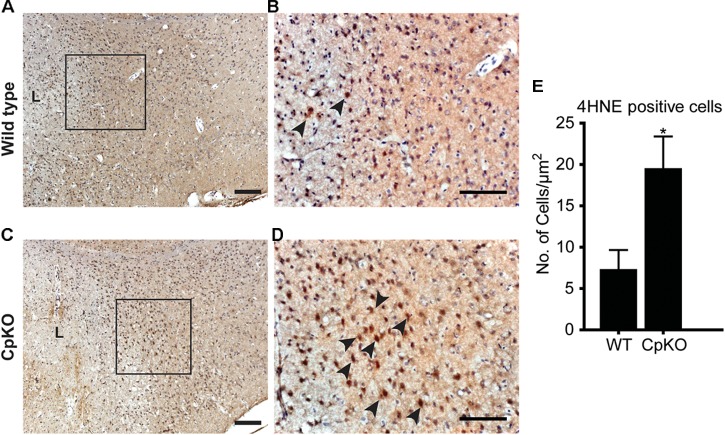
Changes in lipid peroxidation detected by 4-HNE labeling. Tissue sections of wildtype **(A,B)** and CpKO **(C,D)** mice showing immunohistochemical staining for 4HNE. The area demarcated by the black squares in **A** and **C** are shown at higher magnification in panels **B,D**. The lesion area in **A,C** are indicated by “L.” The 4HNE staining is seen as a brown reaction product (arrowheads); the tissue sections were counterstained with Mayer’s hemalaum which labels the cell nuclei blue. **(E)** Graph shows that the number of 4HNE+ cells is significantly higher in CpKO as compared to wildtype mice. Scale bar = 100 μm, ^∗^*p*-value ≤ 0.05.

Two indicators of lipid and protein oxidative damage were assessed. Lipid peroxidation in tissue sections was evaluated by immunohistochemical detection of changes in 4-hydroxynonenal (4HNE). Protein oxidation was assessed by immunohistochemical labeling for 3-nitrotyrosine (3-NT) a product of tyrosine nitration that is mediated by peroxynitrite. Immunohistochemical staining for 4HNE and 3-NT was done on tissue sections of the brain taken at 72 h after pMCAO. The expression of 4HNE was visualized with the DAB technique and appeared as dark brown staining of cells along the border of the infarct region (Figures 7A–D). Adjacent intact regions of the cortex did not show such labeling. The number of 4HNE+ cells in the ipsilateral cortex was significantly higher in Cp null mice as compared to wildtype mice (Figure 7E).

**FIGURE 8:**
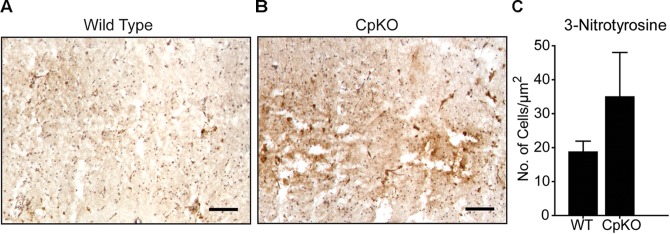
Changes in protein oxidation detected by 3-nitrotyrosine labeling. Tissue sections of wildtype **(A)** and CpKO **(B)** mice showing immunohistochemical staining for 3-NT. The 3-NT staining is seen as a brown reaction product. **(C)** Graph shows that the number of 3-NT+ cells shows a trend to be higher in CpKO compared to wildtype mice but does not reach statistical significance. The tissue sections were counterstained with Mayer’s hemalum which labels the cell nuclei blue. *n* = 4; scale bar = 100 μm.

Immunohistochemistry for 3-nitrotyrosine occurred in the infarct area and there appeared to be a trend to an increase in CpKO mice compared to wildtype mice but this did not reach statistical significance (Figure 8).

### Expression of Inflammatory Cytokines

**FIGURE 9:**
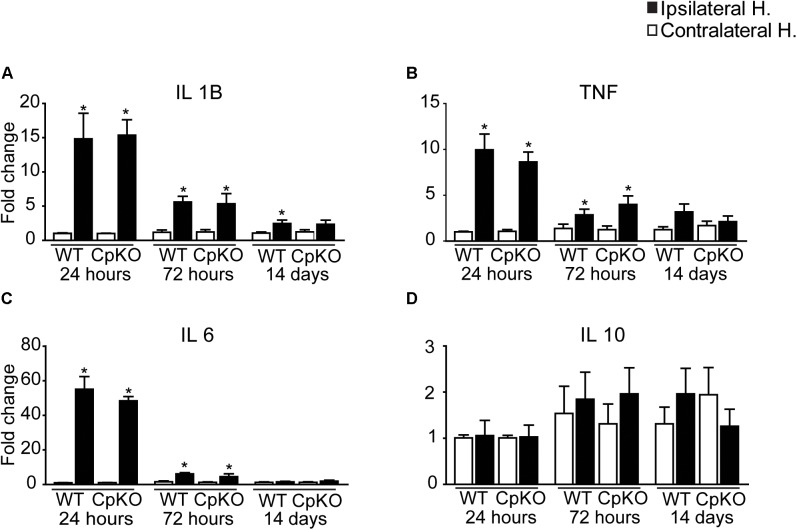
Changes in expression of inflammatory cytokines after pMCAO. The change in the mRNA expression of four key inflammatory cytokines in the brain were assessed by qPCR at 24, 72 h and 14 days after pMCAO in wildtype and CpKO mice. Data are plotted as fold-change on the ipsilateral ischemic side as compared to the uninjured contralateral side. Note the marked increases in IL-1β **(A)**, TNF **(B)** and IL-6 **(C)** at 24 h in both genotypes. They remained elevated at lower levels at 72 h. There were no significant changes in expression of IL-10 **(D)**. ^∗^*p*-value ≤ 0.05.

It is known that the iron status of cells can affect their inflammatory profile (Kroner et al., 2014; Rathore et al., 2012). We therefore assessed whether changes in iron homeostasis after cerebral ischemia also influenced the inflammatory response in Cp null mice. We assessed the expression of four key cytokines that include IL-1β, TNFα, IL-6, and IL-10. Changes in these cytokines was assessed at 24, 72 h, and 14 days after pMCAO. There were marked increases in the mRNA expression of the three proinflammatory cytokines (IL-1β, TNFα, and IL-6) at 24 h, which remained elevated at lower levels at 72 h after pMCAO (Figure 9). No differences were noted between genotypes. The highest increases were detected at 24 h when IL-1β increased by 15-fold, TNFα by 10-fold and IL-6 by about 55-fold. These values dropped markedly by 72 h but were still significantly higher in the lesioned side versus the unlesioned side. By 14 days the values had almost returned to unlesioned control levels (Figure 9). IL-10 levels were not significantly changed but there appeared to be a trend to an increase at 72 h and 14 days. The important point to note here is that the absence of Cp does not alter the expression of key inflammatory cytokines after pMCAO as compared to wildtype mice.

## Discussion

We provide evidence that the lack of Cp leads to loss of functional recovery and greater lesion size after pMCAO in mice. This was accompanied by alterations in expression of molecules involved in regulating iron homeostasis in the CNS, including reduction in expression of iron efflux transporter (ferroportin), increase in iron uptake transporter (DMT1) and increased iron accumulation (ferritin). Iron histochemistry showed deposition of iron in cells along the border of the lesion at early timepoints after pMCAO and throughout the infarct area by 14 days. Double immunofluorescence labeling for ferritin (a surrogate marker for iron) and cell type specific markers revealed that macrophage/microglia and astrocytes along the lesion border accumulate iron. The labeling in astrocytes being greater in Cp null mice. These changes were also accompanied by increased lipid peroxidation (4HNE staining) and greater nitration of tyrosine residues in proteins as evidenced by increased 3-nitrotyrosine labeling. Increases in key inflammatory cytokines in both genotypes early after pMCAO indicate that inflammation contributes to the evolving pathology but is not influenced by the lack of Cp.

This study provides clear evidence that lack of Cp results in larger lesions and worsening of functional recovery as determined by the “sticky-tape” removal test. Wildtype mice take longer time to remove the adhesive tape between 1 and 7 days after pMCAO as compared to sham-control mice, and subsequently return to almost normal values at day 14. This may be due to neural plasticity-mediated reorganization of neural circuits that lead to recovery of function (Carmichael et al., 2001; Arvidsson et al., 2002; Winship and Murphy, 2009). Of interest to note is that function worsens in Cp null mice as compared to wildtype mice at 7 and 14 days, suggesting that the lack of Cp may induce neurogenerative processes during the second week after pMCAO in Cp null mice. This may be related to free radical mediated damage triggered by the absence of the two major functions of Cp, namely (i) its ability as a ferroxidase to safely convert ferrous iron (Fe2+) to its ferric (Fe3+) form; (ii) its ability to partner with Fpn to export iron out of cells (Calabrese et al., 1989; Patel et al., 2002).

Ferrous iron can react with hydrogen peroxide, which is produced in mitochondria through normal cellular metabolism and via the Fenton reaction produce highly toxic hydroxyl radicals that can damage lipids, proteins and DNA (Hentze et al., 2004; Rouault and Tong, 2005). Ceruloplasmin has six domains with three mononuclear type 1 coppers in domains 2, 4 and 6, as well as a trinuclear copper center that lies between domains 1 and 6 (Calabrese et al., 1989; Zaitsev et al., 1999). The mononuclear coppers in domains 4 and 6 participate in the oxidation of ferrous to ferric iron (Zaitsev et al., 1999; Quintanar et al., 2004, 2007). This ferroxidase activity is coupled via electron transfer to the reduction of dioxygen to 2H_2_O at the trinuclear copper center (Calabrese et al., 1989; Zaitsev et al., 1999; Quintanar et al., 2004; Bento et al., 2007). Ceruloplasmin, therefore, safely oxidizes ferrous iron without the generation of free radicals as would happen with the Fenton reaction. Mutations in the trinuclear copper center lead to loss of ferroxidase activity and the degradation of Cp as seen in patients with aceruloplasminemia (Kono, 2013). These patients show iron accumulation in the CNS which increases with age. We have seen increased oxidative stress and iron deposition in the CNS in the Cp null mice with age (Patel et al., 2002; Jeong and David, 2006). On the other hand, iron accumulation occurs because Cp partners with Fpn, the iron efflux transporter, to oxidize ferrous iron that is exported via Fpn (Jeong and David, 2003). In the absence of Cp, Fpn is internalized and degraded (De Domenico et al., 2007), leading to lack of iron efflux and thus iron accumulation. It therefore makes sense that we see significant reduction in the expression of Fpn in the ischemic brain of mice that lack Cp, as well as see increased oxidative damage (4HNE and 3NT) in these mice.

Using ferritin immunofluorescence labeling as a surrogate marker for iron we previously reported that iron accumulation in normally aging Cp null mice occurs in astrocytes, and that the neuronal loss seen may be due to the inability of astrocytes to deliver iron to neurons (Jeong and David, 2006). This may be because unlike neurons which do not directly contact CNS blood vessels, astrocytes are intimately associated with blood vessels via their astrocytic end-feet (Jeong and David, 2006; McCarthy and Kosman, 2015). In other words, astrocytes are equipped to acquire iron from blood vessels in the CNS and deliver it to neurons (McCarthy and Kosman, 2015). After CNS injury, such as spinal cord injury, Cp null mice show greater iron accumulation that is localized to macrophages/microglia at the site of injury (Rathore et al., 2008). These are mainly macrophages that have phagocytosed red blood cells (Rathore et al., 2008). However, in CNS lesions in which hemorrhage does not occur, as in multiple sclerosis or its animal model (EAE), macrophages and microglia accumulate iron likely from phagocytosis of dead and dying cells (Hametner et al., 2013; Zarruk et al., 2015). In the present study, we also see iron accumulation in CD11b+ macrophages/microglia initially around the border of the lesion and later throughout the infarcted area, suggesting that these iron laden cells may be infiltrating macrophages and resident microglia that have phagocytosed dying cells. Interestingly, in Cp null mice, increased ferritin staining was seen in GFAP+ astrocytes lining the lesion border as GPI-Cp is normally expressed selectively by astrocytes in the CNS (Patel and David, 1997). This may add to the iron burden in Cp null mice compared to wildtype mice with pMCAO.

It is not clear at present what might underlie the dysregulated massive 19.5-fold increase in DMT1 protein expression in wildtype mice 72 h after pMCAO. Interestingly, the increase in DMT1 at this time point in Cp null mice was 3 times less (sixfold). The latter might be a way to counteract reduced iron efflux and resultant increase in iron accumulation in Cp null mice due to the reduction in Fpn (50%). The retention of Fpn on the cell surface requires the presence of GPI-Cp (De Domenico et al., 2007). Given that Cp is expressed only in astrocytes in the CNS, the 50% reduction in Fpn seen by Western blot is likely to largely affect astrocytes. In fact, the immunofluorescence labeling experiments showed that there is increased ferritin expression in GFAP+ astrocytes in Cp null mice compared to wildtype mice. So, the lower level of increase in DMT1 in Cp null mice could be a compensatory mechanism that prevents even greater increases in intracellular iron in astrocytes. As mentioned earlier, since the increase in DMT1 in both genotypes is not accompanied by increases in TfR1 expression, it is unlikely that the increased DMT1 is involved in the release of endosomal iron from Tf-TfR1-dependent iron uptake but instead is likely to be involved in Tf-independent cellular iron uptake. We have previously reported that astrocytes express DMT1 on their cell surface but not TfR1, and so can take up iron independent of TfR1 (Jeong and David, 2003) as seen in other non-neural cell types (Wessling-Resnick, 2000).

Inflammation is a well-recognize feature of ischemic stroke (Offner et al., 2006; Wang et al., 2007; Denes et al., 2010; Zarruk et al., 2012; Amantea et al., 2015) in which there is rapid upregulation of pro-inflammatory cytokines. It is important to note that we see a rapid increase in expression in pro-inflammatory cytokines IL-1β, TNFα and IL-6 in both genotypes with no differences between them over the first 14 days after pMCAO. This suggests that the lack of Cp has no influence on the expression of these key inflammatory mediators after ischemic stroke. Recent studies have reported that parenteral treatment with soluble Cp improved lesion size and functional outcome after transient ischemic stroke in mice and rats (Tuo et al., 2017). What we show here is that in addition, the normal levels of Cp present in wildtype mice already has a significant beneficial effect, since lack of this Cp leads to worsening of functional recovery and to larger lesion size.

## Concluding Remarks

This study further elucidates the role of Cp in ischemic stroke. Ceruloplasmin has two fundamental roles (i) in oxidizing ferrous iron and (ii) in the egress of ferrous iron out of cells via Fpn. Lack of Cp, therefore, leads to excess iron buildup in cells. Using wildtype and Cp null mice, we provide clear evidence that Cp has tissue protective effects in the acute phase (24 h) as the ischemic lesion size is larger in Cp null mice. Further evidence of the importance of this antioxidant in the acute phase after pMCAO is suggested by the ninefold increase in its expression at 72 h. In addition, our functional analysis revealed that the absence of Cp leads to worsening of functional recovery at 7 and 14 days after onset of ischemia. This suggests a late effect which could be due to detrimental effects on synaptic reorganization and circuitry via free-radical mediated effects on neurons, astrocytes and microglia. Future work will need to address if Cp influences microglial and astrocyte responses after ischemic stroke that can impact on functional recovery. Analysis of dendritic arborizations and dendritic spines in the penumbral regions of the cortex in wildtype and Cp null mice will also be worth assessing. Evidence of increased iron in the ischemic tissue was detected by iron histochemistry. Furthermore, immunofluorescence staining for ferritin (a surrogate marker for iron) showed increased ferritin in both astrocytes and macrophages/microglia after pMCAO in wildtype mice but this is significantly higher in astrocytes in Cp null mice. These perturbations in iron levels in the tissue was accompanied by increased oxidative damage to lipids and proteins. There is evidence that systemic treatment with Cp improves outcome after ischemic stroke in rodents. Our current study shows that the increased expression of Cp in the ischemic cortex of wildtype mice has important neuroprotective effects after cerebral ischemia.

## Author Contributions

JZ, FR, and SD planned the study and interpreted the results. JZ, FR, and LL carried out the experiments. JZ and FR analyzed the data and edited the manuscript. SD wrote the manuscript.

## Conflict of Interest Statement

The authors declare that the research was conducted in the absence of any commercial or financial relationships that could be construed as a potential conflict of interest.
